# The association between sleep duration, insomnia and sleep patterns with metabolic syndrome: an integrative review

**DOI:** 10.3389/fcdhc.2026.1794920

**Published:** 2026-04-16

**Authors:** Bekalu Bewket, Adriano Marçal Pimenta

**Affiliations:** 1 College of Medicine and Health Science, Injibara University, Injibara, Ethiopia; 2 Posgraduate Program in Nursing, Universidade Federal do Paraná, Curitiba, Brazil; 3 Department of Nursing, Universidade Federal do Paraná, Curitiba, Brazil

**Keywords:** daytime napping, insomnia, integrative review, metabolic syndrome, sleep duration

## Abstract

**Introduction:**

Due to the use of various methods across different studies, the association between sleep duration, insomnia and sleep pattern with metabolic syndrome (MetS) remains inconsistent. Integrative reviews that thoroughly synthesize data from various study types are therefore required in order to clarify the relationships between particular aspects of sleep and MetS. Therefore, this integrative review aimed to assess and summarize the most recent data regarding the relationship between sleep duration, sleep patterns and insomnia with MetS.

**Design:**

Integrative review.

**Methods:**

Problem identification, literature search, data evaluation, data analysis, and presentation five stage methodology developed by Whitmore and Knaff’s (2005) guided this review. Four databases including; PubMed, Embase, Scopus and Web of Science were utilized. The quality of included studies were assessed by the Mixed Methods Appraisal Tool (2018). Conventional quantitative content analysis method was used to analyze extracted date and the preferred reporting items for systematic reviews and meta analyses checklist guided the reports of this review.

**Result:**

Initially a total of 25,472 articles were retrieved, 12 cross-sectional, 5 cohorts, 1 case-control studies were finally included. This study revealed sleep duration, insomnia and specific sleep characteristics such as; long daytime napping > 90 minutes, irregular sleep, social jetlag and early wake-up linked with increased risk of MetS.

**Conclusion:**

MetS is a complex problem and could be affected by the interaction of multiple sleep characteristics. Short sleep duration consistently was associated with MetS. However, the association between long sleep duration and insomnia with MetS remained inconsistent.

## 1 Introduction

Metabolic syndrome (MetS) is a collection of interconnected cardio metabolic risk factors, including central obesity, hypertension, dyslipidemia (high triglycerides and low HDL cholesterol), and impaired glucose metabolism (1).

The first definition, which was Insulin resistance syndrome (IR) was proposed by World Health Organization (WHO) in 1998 (2). Several criteria have been proposed by different organizations since the first diagnosis. In 2005 the International Diabetes Federation (IDF) proposed the new universally applicable diagnosis of MetS with obesity as a central component, and must be assessed using age-specific and ethnicity/race-specific cut of values for waist circumference (3). In addition to this the American Heart Association (AHA)/National Heart, Lung and Blood Institute (AHA/NHLBI) (4) and the American Association of Clinical Endocrinologists (5) each have introduced their own definition. The harmonized definition criteria for MetS was proposed from a collaborative statement by IDF, AHA/NHLBI, the World Heart Federation, the International Atherosclerosis Society and the International Association for the Study of Obesity in 2009 (6). Above all the National Cholesterol Education Program-Third Adult Treatment Panel (NCEP-ATP III) has been the most widely used definition for MetS (7).

The prevalence of MetS in adult populations around the world ranging from 20% to 30%, it poses a serious global public health concern (8). People with MetS have a significantly higher risk of several debilitating conditions, including kidney disorders, type 2 diabetes, atherosclerosis, cardiovascular disease, cancer, and premature mortality in general population (9–12).

Even though genetic predisposition, diet, and physical inactivity are known factors, new research shows that sleep patterns are a significant but little-known determinant of metabolic health (13). MetS is widely acknowledged as a multifactorial disorder closely associated with lifestyle factors, such as poor sleep hygiene and short sleep duration (14–17). Moreover the association had also been linked with long sleep duration (18). However, a few studies showed a U-shaped relationship between sleep duration and MetS and its components (19, 20).

The biological process of sleep has several dimensions, including timing, quality, duration, and continuity. Cardio metabolic dysfunction has been linked to sleep disturbances, including those that are brief or excessively prolonged, have poor subjective quality, occur at irregular times, or are caused by conditions like insomnia (21).

Acute and long-term sleep deprivation has been shown in experiments to cause insulin resistance, sympathetic nervous system activation, elevated cortisol levels, and changes in hormones that regulate appetite, including ghrelin and leptin (22, 23). These pathophysiological alterations offer believable explanations for how sleep disruption could aid in the development of MetS.

Epidemiological evidence indicates that both sleep duration and insomnia are significantly linked to the development of MetS. A meta-analysis of multiple cross-sectional studies demonstrated that individuals with short sleep duration (typically <6.5 hours) have a higher likelihood of MetS compared with those sleeping between 7 and <8 hours (24). Notably, many of these studies also identified an elevated risk of MetS among individuals reporting long sleep duration (>9 hours) (25–28). In addition, recent meta-analytic findings show that poor sleep quality is likewise associated with an increased risk of MetS (29).

While the evidence for long sleep is less clear, epidemiological studies have consistently connected short sleep duration to obesity, impaired glucose tolerance, and hypertension (30, 31). Both short (less than six hours) and long (more than nine hours) sleep durations were linked to an increased risk of MetS, according to a meta-analysis study, indicating a U-shaped relationship (31). Additionally, cohort studies show that incident MetS is predicted by consistently inadequate sleep over a number of years, especially for women of reproductive age (32). Even after controlling for sleep duration, poor sleep quality and fragmentation have been linked to higher risks of central obesity and insulin resistance (33). Furthermore, circadian misalignment, like that which occurs in night shift workers, throws metabolic homeostasis off balance and is linked to negative glucose and lipid profiles (34).

Findings are still inconsistent despite mounting evidence because of variations in study design, subjective versus objective sleep assessment techniques, and population characteristics. Furthermore, the majority of research is cross-sectional, which restricts the ability to draw conclusions about causality. Integrative reviews that thoroughly synthesize data from various study types are therefore required in order to clarify the relationships between particular aspects of sleep and MetS. Such synthesis is essential to guide future research focused on interventions as well as clinical practice, where sleep assessment may be a modifiable risk factor.

The purpose of this integrative review was to assess and summarize the most recent data regarding the relationship between sleep duration, sleep patterns and insomnia with MetS. We aim to provide a thorough understanding of the role of sleep in metabolic health, highlight mechanistic pathways, and identify gaps in the literature to direct future research by looking at sleep duration, patterns, and insomnia in relation to MetS.

## 2 Methods

### 2.1 Design

The integrative review method developed by Whittemore and Knafl was utilized in this study to examine and compile diverse research methods on sleep patterns, duration, insomnia and their correlation with MetS in the general adult population (35).

The integrative review approach made it possible to take into account empirical studies about MetS and sleep patterns. Problem identification, literature search, data evaluation, data analysis, and presentation are the five steps in Whittemore and Knafl’s methodology for data collection, analysis, and synthesis.

Preferred Reporting Items for Systematic Reviews and Meta analyses (PRISMA) checklist guided the conduct and reporting of this review (36).

#### 2.1.1 Problem identification

The research problem for this study was defined according to the following question: What are the scientific evidences regarding the relationship between sleep patterns, duration, insomnia with MetS?

#### 2.1.2 Literature search

We performed a total search to find studies of sleep duration, sleep pattern/insomnia and MetS. A search of the following databases was conducted to retrieve articles published from 2010 to 2025 with a combination of medical subject heading and entree terms, supplemented with free-text words: PubMed, Embase, Scopus and Web of Science.

Search terms were (Sleep OR “sleep patterns” OR “sleep duration” OR insomnia OR “sleep quality” OR “sleep disturbance” OR “sleep disorders” OR “circadian rhythm”) AND (“metabolic syndrome” OR “Metabolic Syndrome X” OR “syndrome X” OR “cardio metabolic risk” OR “cardio metabolic syndrome”).

Only English-language human research was taken into account. By looking through the reference lists of the included research and pertinent reviews, more sources were found.

Eligible studies included adult people; evaluated sleep duration, sleep pattern or insomnia as the main exposure; and reported MetS as an outcome.

Cross-sectional, case-control, and cohort observational studies that reported pertinent outcomes were among the designs that qualified (**Table 1**).

**Table 1:** Characteristics and summary of included studies in the integrative review on sleep duration, sleep pattern, insomnia and its association with metabolic syndrome.

Author/year/country	Aim	Sample size and population	Study design	Measurement tools	Results	Implications and suggestions
(37), Korea	To investigate the associations of various sleep characteristics with the status and incidence of metabolic syndrome (MetS) in middle-aged Koreans	Eligible participants aged 30-55 years (the final analytical sample consisted of 1,984 participants at baseline and 1,216 participants at the follow-up)	Cohort study (with cross-sectional and longitudinal analysis)	MetS was defined based on National Cholesterol Education Program criteria (NCEP/ATP III); Pittsburgh Sleep Quality Index (PSQI). Night sleep duration (hours) was categorized as recommended (7 to ≤9 hours, reference), 30 short (6 to < 7 hours), and very short (< 6 hours).	After follow-up, 15% belonged to MetS groups. MetS was associated with long sleep latency (prevalence ratio - PR 1.33, 95% confidence interval - 95% CI 1.03-1.73) and early wake time (< 6:00; PR 1.29, 95% CI 1.01-1.63); Longitudinal analysis of participants without MetS at baseline indicated a significant increase in MetS risk associated with very short sleep duration (< 6 hours; hazard ratio - HR 1.72, 95% CI 1.06- 2.79), long sleep latency (> 30 minutes; HR 1.86, 95% CI 1.10-3.12), and early wake time (< 6:00 o’clock; HR 1.73, 95% CI 1.01-2.97).	Both insufficient and excessive sleep may elevate early MetS risk; promoting optimal sleep in youth is critical
(39), Iran	To investigate the association between night sleep of less than 7 hours and insomnia with MetS and its components in health-care workers	410 health-care workers (23 to 63 years-old) in an educational hospital	Cross-sectional study	The insomnia severity index (ISI) questionnaire was utilized in this study to evaluate insomnia and its symptoms; Self-reported sleep duration was also utilized and stratified as short sleep (< 7 h/night), normal sleep (7 to 8 h/night), long sleep (≥ 9 h/night); MetS was determined using the NCEP/ATP III.	124 individuals (30.24%) met the criteria for the MetS; Individuals who had the MetS were considerably more likely to be male (50.8% vs. 31.81%); The mean insomnia score based on the ISI questionnaire was 11.4 ± 5.7 with a range of 0-24; The prevalence of MetS was significantly higher among workers with insomnia than among those without insomnia after adjusting age and sex (model 1) (*p* <.001; OR=2.77; 95% CI 2.12-3.98); In addition, the prevalence of MetS was significantly higher among workers with short sleep duration than among those with normal sleep duration (*p* < 0.001; OR=2.37; 95% CI 1.29-2.68).	Workplace sleep hygiene and periodic screening for insomnia recommended to reduce occupational MetS risk
(Kyung et al., (40)), USA	To explore the relationship between shift work, sleep duration, social jetlag, and the risk of MetS among U.S. workers and the moderating effect of sleep duration and social jetlag on this relationship	4,136 U.S. workers (≥ 18 years-old) from the National Health and Nutrition Examination Survey (NHANES) in 2017–2020	Cross-sectional study	Self-reported sleep duration was categorized into three groups: (1) short sleep duration (less than 6 h), (2) normal sleep duration (6 h to less than 9 h), and (3) long sleep duration (9 h or more); Social jetlag was calculated, and the absolute difference between mid-point sleep duration on weekdays and weekends was calculated; Social jetlag was also categorized into three groups: (1) normal social jetlag (less than 1 h), (2) moderate social jetlag (1 h to less than 2 h), and (3) severe social jetlag (2 h or more); MetS defined by NCEP/ATP III criteria.	53.3% had MetS, with a higher proportion of shift workers (63.8% vs. 56.7%) and those sleeping less than 6 h or more than 9 h per day (22.3% vs. 19.1%, *p* = 0.044) in the affected group; Shift workers were initially found to have an increased risk of metabolic syndrome (PR = 1.03, 95% CI: 1.02, 1.17); However, this association was mitigated when accounting for the interaction with social jetlag; Specifically, 1 to <2 h of social jetlag interacted significantly, increasing MetS risk (PR = 1.16, 95% CI: 1.09, 1.25), whereas 1 to <2 h alone showed a protective effect (PR = 0.90, 95% CI: 0.84, 0.94).	These findings suggest that optimizing sleep schedules and addressing social jetlag may be crucial in mitigating MetS risks
(Chaudhry; Brian; Morrell, (41)), USA	To examine the relationship between sleep duration and metabolic syndrome severity scores (MSSS) in a sample of emerging adults	Convenience sample of 3,816 student participants between the ages of 18 and 24 years, enrolled in a general education, introductory nutrition course	Cross-sectional study	MetS was defined based on National Cholesterol Education Program criteria (NCEP/ATP III); Sleep duration was calculated from the difference in self-reported bedtime and wake time acquired through an online survey.	MetS (*≥*3 criteria) was present in 3.3% of students. Mean MSSS was *-*0.65 *±* 0.56, the reported sleep duration was 8.2 *±* 1.3 h/day; MSSS was higher among low sleepers (<7 h/day) and long sleepers (>9 h/day) compared to the reference sleepers (7–8 h/day) (*-*0.61 *±* 0.02 and *-*0.63 *±* 0.01 vs. *-*0.7 *±* 0.02, respectively, *p* < 0.01)	Short sleep is a causal risk factor for MetS; interventions should promote adequate sleep duration
(Elshoeibi et al., (42)), Qatar	To examine the link between short sleep duration and MetS	Qatar Biobank, with 1,000 adult participants (≥ 18 years-old)	Case-control study	The duration of sleep was assessed using a self-reported questionnaire; WHO criteria used to diagnose MetS.	There was a higher proportion of individuals with MetS in the short sleep duration group compared to the normal sleep duration group (22.8% vs 15.8%, respectively); The good sleep group had a higher proportion of individuals with no risk factors for MetS compared to controls (18.6% vs. 11.0%, respectively); Individuals with MetS had almost 2-fold greater chance of short sleep duration when compared to those without risk factors for MetS (Odds Ratio - OR 1.91, 95% CI: 1.14-3.20, *p* = 0.014).	Short sleep duration is a significant predictor of MetS in Qatar, particularly for males; Weight control interventions may mitigate risk
(Zhang et al., (43)), China	To investigate whether sleep pattern is associated with MetS among young adults	1,151 students from College Student Behavior and Health Cohort Study	Cohort study (with cross-sectional and longitudinal analysis)	Metabolic scores were calculated using four metabolic parameters including BMI, WC, FBG, and insulin, total score ranges from 0 to 4, and the higher the score the sever metabolic score; Pittsburgh Sleep Quality Index (PSQI) 0 to 3 scale used to assess Excessive daytime sleepiness; Insomnia Severity Index (ISI) was used to assess insomnia symptoms with a total score ranging from 0 to 28 and with a score of ≥9 defined as insomnia and <9 defined as no insomnia; Sleep duration was reported as the number of hours spent sleeping during the day (including naps); Chronotype was evaluated from rMorning and Evening Questionnaire (rMEQ) categorized as 1) definitely a ‘morning’ person; 2) more a ‘morning’ person than ‘evening’ person; 3) more an ‘evening’ person than a ‘morning’ person; 4) definitely an ‘evening’ person.”).	In the baseline survey, we found that a total of 41 (4.1%) participants had poor sleep patterns; Metabolic scores were significantly higher among college students with poor sleep patterns, compared with those who with healthy sleep patterns at baseline (1.00 ± 0.96 vs. 0.78 ± 0.72, p < 0.05) and 2- year follow-up (0.34 ± 0.65 vs. 1.50 ± 1.64, p < 0.05); After covariates were adjusted by multivariate linear regression, poor sleep pattern (β = 0.22, 95% CI: 0.06-2.53, p = 0.001) was associated with elevated metabolic scores at the 2-year follow-up.	Poor sleep patterns in college students associated with increased risk of MetS, therefore promoting healthy sleep behaviors in college students may minimize metabolic risks
(Belayneh et al., (44)), Ethiopia	To measure the prevalence of MetS and to identify specific risk factors among adult populations who visited Dessie Comprehensive Specialized Hospital (DCSH), Ethiopia	419 adults attending Dessie Comprehensive Specialized Hospital	Cross-sectional study	Self-reported sleep duration was reported with the following category responses: *<* 6 hrs., 6 to7 hrs., 8 to 9 hrs., and 10 hrs.; The recommended sleep duration ranges from 6 hrs. to 9 hrs. for adults as defined by National Sleep Foundation of America; MetS was classified based on harmonized definition of joint interim statement of IDF and NCEP/ATP III.	The proportion of MetS among adults who attended DCSH was 35.0% [95% CI, (30.5, 39.5)] The odds of MetS among adults with sleeping duration less than six hours per day was about five times higher than the odds of MetS in adults who had a sleeping duration often and more hours per day [OR: 4.62; 95% CI: (1.02, 20.98)].	Short sleep duration less than 6 hours was found to show increased risk for MetS; This finding highlight having average sleep is essential to promote metabolic health
(Ogura et al., (45)), Japan	To examine the relationship between subjective sleep irregularity and MetS	3,880 participants from Japan Multi-Institutional Collaborative Cohort Study (J-MICC study)	Cross-sectional study	MetS was defined according to Japanese criteria; The average self-reported sleep duration was categorized based on the tertile value: *<*6h/day, T1; 6–*<*7h/day, T2; and ≥7 h/day, T3; Self-reported sleep regularity was reported regular/irregular.	The irregularity of sleep was significantly associated with MetS (OR 1.231, 95% CI 1.101-1.375) adjusted for age, sex, METs, sleep duration, bedtime, drinking and smoking statuses, and a history of using sleeping pills.	This study showed that irregular sleep is more strongly associated with MetS than sleep duration or bedtime
(Peila et al., (46)), USA	To examine the association of sleep duration and insomnia with MetS and its components using longitudinal data from the Women’s Health Initiative (WHI)	5,159 postmenopausal women (50-79 years-old)	Cohort study (with cross-sectional and longitudinal analysis)	Sleep duration was self-reported as hours/night (≤6, 6- < 7, 7- < 8, 8- < 9, 9- < 10, ≥10); Insomnia Rating Score (WHIIRS (5 item and 4 scale instruments with the highest total score of 20); Insomnia was defined as a score ≥9; MetS definition for women proposed by NCEP/ATP-III.	In cross-sectional analysis, baseline sleep duration ≥9 h was positively associated with MetS (OR=1.51; 95% CI 1.12–2.04); Insomnia had a borderline positive association with MetS (OR=1.14; 95%CI 0.99-1.31); In the analysis stratified by baseline insomnia status, there were positive associations between long sleep duration (≥9 h) and MetS in both strata of insomnia (OR≥9 h/insomnia=2.89; 95% CI 1.39-6.01 and OR ≥9 h/no-insomnia=1.54; 95% CI 1.03-2.00 respectively); In the longitudinal analysis, change from restful sleep to insomnia over time was associated with increased chance of developing MetS (OR=1.40; 95% CI 1.01–1.94).	Suggests early screening for sleep disorders may prevent cardiometabolic complications in older women
(Fan et al., (19)), China	To evaluate the prevalence of MetS and explore the association between sleep duration and MetS	8,272 adults aged ≥ 18 years-old	Cross-sectional study	MetS was defined according to Chinese guideline for type 2 diabetes; Self-reported sleep duration was classified as <6, 6-9, and > 9 hours.	The estimated prevalence of MetS was 30.3% (95% CI, 29.3 to 31.3); Men were more likely to have MetS than women (*p* = 0.01); There was a U-shaped relationship between sleep duration and MetS and its components; sleep duration <6hours or >9hours were associated with higher risk of MetS (OR from 1.10 to 2.15).	High prevalence of MetS in men group and increased metabolic risk among both short and long hour sleepers indicates the importance of regular screening and optimal sleep recommendations are important particularly for men population
(47), Sweden	To study the relationship between insomnia disorder and MetS in the Swedish CArdioPulmonary bioImage Study (SCAPIS) pilot cohort	830 participants, aged 50-64 years, stratified by low and high socioeconomic residential area	Cross-sectional study	MetS was classified using NCEP/ATP III; The insomnia severity index (ISI) questionnaire was used for classification of insomnia (cut-off: ISI score >=15).	MetS was found in 17.1% and insomnia in 12.4% of the population The prevalence of MetS was 24.3% and 16.1% in the insomnia and non-insomnia groups, respectively (P = 0.039); Insomnia was associated with an approximately two-fold increased risk of MetS (OR 1.97, 95% CI 1.00–3.86, *p* = 0.049).	Screening for insomnia and treating it will reduce the risk of MetS
(Gaston et al., (48)), USA	To investigate the cross-sectional associations between multiple subjective sleep characteristics and having ≥3 prevalent metabolic abnormalities consistent with MetS among White, Black, and Hispanic/Latina women	38,007 eligible women (13,988 premenopausal, 24,019 postmenopausal), 35 to 74 years-old	Cross-sectional study	Self-reported sleep duration categorizing average sleep duration as short (< 7 h) and recommended (7-9 h); Participants reported napping frequency, dichotomized as frequent napping ≥3 naps per week vs. < 3 naps per week; Inconsistent weekly sleep patterns determined (yes vs. no); Insomnia symptoms included either difficulty falling asleep (taking ≥30 min vs. < 30 min to fall asleep on average) or difficulty staying asleep (awakening ≥3 times per night/day, ≥3 nights/days per week vs. awakening < 3 times per night/day and/or < 3 nights/days per week) vs. neither; Cumulative sleep score calculated as short sleep duration, inconsistent weekly sleep patterns, frequent napping, and insomnia symptoms [range: 0–5]); MetS was classified based on harmonized definition of joint interim statement of IDF and NCEP/ATP III.	Among both premenopausal and postmenopausal participants, black and Hispanic/Latina women had similarly higher prevalence of inconsistent weekly sleep patterns, sleep debt, frequent napping, insomnia symptoms, and both short sleep and insomnia symptoms compared to white women; Compared to premenopausal women, postmenopausal women also had higher prevalence of MetS (white-14% vs. 4.6%, black-28% vs. 12%, and Hispanic/Latina-21% vs. 8.7%); Women with prevalent MetS generally had a higher prevalence of poor sleep characteristics regardless of race/ethnicity compared to women without MetS; Associations between certain poor sleep characteristics, including short sleep duration, insomnia symptoms, difficulty staying asleep, concurrent short sleep duration and insomnia symptoms, and MetS were stronger among premenopausal compared to postmenopausal women; Associations between certain poor sleep characteristics [i.e., short sleep (PR premenopausal=1.23, 95% CI 1.06-1.42; PR postmenopausal=1.09, 95% CI 1.02-1.16], and insomnia symptoms (PR premenopausal=1.21, 95% CI 1.05-1.41], PR postmenopausal=1.11, 95% CI 1.05-1.18], and prevalent MetS were stronger among premenopausal compared to postmenopausal women.	Promotion of good sleep hygiene like acquiring the recommended amount of sleep and addressing insomnia symptoms throughout the life course may be a worthwhile approach to the prevention of poor cardiometabolic health among women, especially prior to the menopausal transition
(Smiley; King; Bidulescu, (49)), USA	To assess the association of sleep duration and MetS prevalence in the National Health and Nutrition Examination Survey (NHANES) 2013/2014	Sample size included 2,737 adults (≥ 18 years-old) in the (NHANES)	Cross-sectional study	Metabolic syndrome was defined when 3 or more of the findings were present according to NCEP ATPIII criteria; Self-reported sleep duration was utilized (continuous variable)	About 31.5% had MetS; The mean (SD) duration of sleep was 6.82h (1.22h); There was a U-shaped association between sleep duration and MetS – generalized additive model - GAM (effective degree of freedom - EDF = 2.03, *p* = 0.20); The lowest risk of MetS was observed in people sleeping 7 hours/night; There was a significant U-shaped association between sleep duration and MetS severity score in multivariable GAM (EDF = 2.94, *p* = 0.0004); Similarly, the lowest mean MetS severity score was observed in people sleeping 7 hours/night.	Optimum sleep hours approximately 7 hrs. per day is essential for preventing cardio metabolic disease
(Kim et al., (18)), Korea	To examine the association between sleep duration and MetS occurrence among Koreans age 40-69 year olds (HEXA study)	A total of 133,608 participants (44,930 men, 88,678 women) included (40-69 years-old)	Cross-sectional study	Self-reported daily sleep duration including napping was assessed and classified by 4 category responses: < 6 h, 6 to < 8 h, 8 to < 10 h, ≥10 h; MetS was defined using NCEP/ATP III criteria.	About 10.9% of men and 12.7% of women slept less than 6 h, 1.5% of men and 1.7% of women slept greater than 10 h; The overall prevalence of MetS was 29.1% men and 24.5% women; Compared with individuals sleeping 6 to < 8 h per day, less than 6 h of sleep was associated with MetS (OR: 1.12, 95% CI: 1.05–1.19), also greater than 10 h of sleep was associated with MetS (OR 1.28, 95% CI 1.08–1.50).	Reinforces U-shaped sleep-MetS relationship; emphasizes optimal 7–8 h sleep for prevention
(Deng et al., (50)), Taiwan	To investigate the impact of sleep duration on five MetS components in a healthy adult cohort	A total of 162 121 adults aged 20–80 years (men 47.4%) of the MJ Health Database	Prospective cohort study	Self-reported sleep duration was reported in the three categories of sleep duration; that is, “<6 hours/day,” “6–8 hours/day,” and “>8 hours/day”; According to the diagnostic criteria by the American Psychiatric Association, participants were considered as having insomnia symptoms if they reported one or more of the symptoms; MetS was classified based on harmonized definition of joint interim statement of IDF and NCEP/ATP III.	Among participants, 18.6% were short sleepers (<6 hours/day), 72.8% were regular sleepers (6–8 hours/day, referent), and 8.6% were long sleepers (>8 hours/day); More than half of the participants (57.6%) reported that they had insomnia symptoms; Short sleepers were more likely to develop MetS (adjusted HR 1.09 [95% CI 1.05–1.13]); Sleep duration longer than 8 hours was associated with a decreased risk of MetS (adjusted HR 0.93 [95% CI 0.88–0.99]).	Sleep duration may be a significant determinant of metabolic health
(Wang et al., (51)), China	To assess the association between insomnia and metabolic syndrome among different-aged groups	8,017 participants aged 18-82 years	Cross-sectional study	MetS was diagnosed according to the 2005 IDF definition; Insomnia was assessed by the Athens Insomnia Scale, 8 items each item 0-3 scales (AIS); the total score is from 0 to 24 points; participants with scores of 6 and above are considered to have insomnia.	The prevalence of MetS was 29.70% among all participants, 34.61% in males and 24.24% in females; Insomnia (AIS ≥ 6) was found in 1,019 participants (12.71%), of which 366 (4.57%) were diagnosed with MetS and 653 (8.15%) were not diagnosed with MetS (*p <*0.05); Insomnia was not independently associated with MetS across all subjects; however, the association between insomnia and MetS was statistically significant in the male group (OR: 1.35, 95% CI: 1.02–1.77) and the middle-aged group (OR: 1.40, 95% CI: 1.09–1.79), but not in the female group, the young adult group or the older group.	An independent association between insomnia and MetS in males and middle-aged participants, which suggests that treatment for insomnia will contribute to the prevention of MetS in males and the middle-aged population
(Wu et al., (52)), China	To examine the association between daily sleep duration and MetS and its components in middle-aged and older Chinese adults using data from the Dongfeng-Tongji Cohort study	25,184 participants of Dongfeng-Tongji Cohort study (mean age 63.6 years-old), retired employees	Cross-sectional study	International Diabetes Federation (IDF) definition was used to classify MetS; Self-reported daily sleep duration was computed by summing up nighttime sleep duration with daytime napping duration, categorized it into five categories: <7 hours, 7–7.9 hours, 8–8.9 hours, and 9–9.9 hours and ≥10 hours.	Of the participants, 8,046 (31.9%) had MetS; Female participants with longer daily sleep duration (≥8 hours, all *p* < 0.05) per day had a higher risk of MetS compared with those sleeping 7–7.9 hours, adjusting for potential confounders; Longer duration of daytime napping (≥90 minutes, *p* < 0.05) was associated with the risk of MetS in females; Moreover, female participants with longer duration of daytime napping had a higher prevalence of MetS compared with those who did not nap; Daily sleep duration, nighttime sleep duration and daytime duration had no effects on the prevalence of MetS in males; However, nighttime sleep duration was not associated with the risk of metabolic syndrome in either males or females.	Suggests that excessive daytime napping-not nighttime sleep-may elevate MetS risk; gender-specific sleep recommendations needed
(Troxel et al., (53)), Russia	To explore the association between commonly reported sleep symptoms and the development of the MetS prospectively	812 participants from Participants were from the community-based Heart Strategies Concentrating on Risk Evaluation study	Prospective cohort study	MetS was defined based on NCEP/ATP III; Insomnia was diagnosed by Insomnia Symptom Questionnaire (ISQ) with individuals who endorsed the symptom ≥ 3 times per week were coded as 1 (Insomnia) and others coded as 0 (Not).	Over the 3-year follow-up period, 14% of the sample (n = 115) developed the MetS; Specific symptoms of insomnia (difficulty falling asleep [DFA] and “unrefreshing” sleep), but not a syndrome definition of insomnia, were significant predictors of the development of MetS.	Unrefreshing sleep and difficulty of falling sleep both main symptoms of insomnia have been found to predict MetS, this implies that comprehensive assessment of sleep characteristics in metabolic health screening may help earlier identification and management of metabolic disease

Research that only examined other sleep constructs such as obstructive sleep apnea, sleep quality, circadian rhythm, or chronotype without providing independent information on duration or insomnia was disqualified. Case reports, editorials, commentary, narrative reviews, and animal studies that lacked primary data were also disqualified.

Two reviewers independently screened titles, abstracts, and entire texts; discrepancies were settled by discussion. A total of 25,472 articles were first screen for duplication, after which some duplicated articles were removed. 12,321 articles were evaluated for relevance based on titles and abstracts. 12,112 articles removed for wrong population, published in other languages and full length not available. The remaining 209 studies, were assessed and 191 studies were excluded for not being empirical studies and focused on other sleep problems such as sleep apnea. Finally, 18 studies meet the inclusion criteria and included for integrative review.

#### 2.1.3 Data evaluation

The Mixed Methods Appraisal Tool (MMAT, 2018 version) (54), which enables the simultaneous evaluation of qualitative, quantitative, and mixed-methods research within a unified framework, was used to evaluate the methodological quality of all included studies.

Five fundamental design-related criteria were used to evaluate the studies: the validity of the findings, the suitability of the data collection techniques, the completeness of the outcome data, the consideration of confounders, and the clarity of the study objectives. Every criterion was given a rating of “Yes”, “No”, or “Cannot tell”, and each study was given a quality score (**Table 2**).

**Table 2:** Quality assessment of included studies using MMAT criteria in the integrative review on sleep duration, sleep pattern, insomnia and its association with metabolic syndrome.

Study Design	Author/Year	Are the participants representative of the target population?	Are measurements appropriate regarding both the outcome and intervention (or exposure)?	Are there complete outcome data?	Are the confounders accounted for in the design and analysis?	During the study period, is the intervention administered (or exposure occurred) as intended?
Cohort	(Baek et al., (37))	Y	Y	Y	Y	Y
	(Zhang et al., (43))	Y	Y	Y	Y	Y
	(Peila et al., (46))	Y	Y	Y	Y	Y
	(Deng et al., (50))	Y	Y	Y	Y	Y
	(Troxel et al., (53))	Y	Y	Y	Y	Y
Case-control	(Elshoeibi et al., (42))	Y	Y	Y	Y	Y
Cross-sectional	(Kabir-Mokamelkhah et al., (39))	Y	Y	Y	Y	Y
	(Kyung et al., (40))	Y	Y	Y	Y	Y
	(Chaudhry; Brian; Morrell, (41))	Y	Y	Y	Y	Y
	(Belayneh et al., (44))	Y	Y	Y	Y	Y
	(Ogura et al., (45))	Y	Y	Y	Y	Y
	(Fan et al., (19))	Y	Y	Y	Y	Y
	(Zou et al., (47))	Y	Y	Y	Y	Y
	(Gaston et al., (48))	Y	Y	Y	Y	
	(Smiley; King; Bidulescu, (49))	Y	Y	Y	Y	Y
	(Kim et al., (18))	Y	Y	Y	Y	Y
	(Wang et al., (51))	Y	Y	Y	Y	Y
	(Wu et al., (52))	Y	Y	Y	Y	Y

Findings on sleep duration, insomnia, and MetS could be systematically synthesized while taking methodological rigor into account thanks to this technique, which made it easier to evaluate disparate evidence in a consistent and transparent manner.

#### 2.1.4 Data analysis

Considering the final included studies were quantitative, but heterogeneous in design (cross-sectional, case-control, cohort) a narrative synthesis method was employed in line with Whittemore and Knafl integrative framework (35).

Conventional quantitative content analysis was used to assess patterns in the association between sleep duration, sleep patent and insomnia with MetS.

The fifth step of Whittemore and Knafl’s methodology (35), presentation (2.1.5), was displayed in a section of results.

Results were coded as positive association, negative association, no association, diagnostic criteria for MetS and measurement instruments for sleep characteristics.

##### 2.1.4.1 Terms

Sleep duration: The total amount of time an individual stayed asleep in 24 hours, with a recommended 7 to 9 hours of sleep for an adult (55).

Insomnia: A persistent difficulty with sleep initiating, duration, consolidation, quality, occurring despite adequate sleep opportunity and resulting daytime dysfunction (56).

Sleep pattern: In this study sleep pattern includes (long sleep latency, early wake-up, longer duration daytime napping, irregular sleep and social jetlag).

*Long sleep latency*: Sleep latency is a measure of time it takes to enter sleep, a normal sleep latency value for a health adult is between 10 to 15 minutes (57).

*Long duration daytime napping*: A nap is a short sleep, typically taken during daylight hours and it usually recommended ranging from 20 to 30 minutes and long daytime napping longer than 60 minutes associated with adverse health outcomes (58).

*Social jet-lag*: The term social jetlag (SJL) refers to a form of circadian misalignment that arises from the discrepancy between activity/sleep schedules on school/work days and free days. SJL defined as the absolute difference between the midsleep point on free days (MSF) and the midsleep point on work (or school) days (MSW) (59).

*Irregular sleep*: is characterized by the relative absence of a circadian pattern in an individual’s sleep–wake cycle (60).

## 3 Results

### 3.1 Selection of the studies

**Figure 1:**
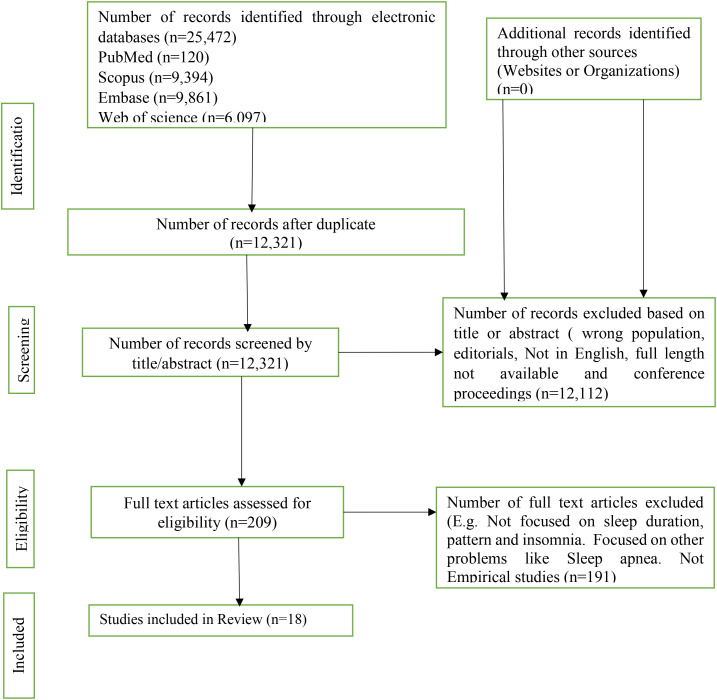
PRISMA flow diagram of literature search.

Initially a total of 25,472 articles were identified from the databases. After title, abstract and full article screening and removing duplicates, eighteen articles meet the eligibility criteria and were included in the final synthesis (**Figure 1**).

### 3.2 Characteristics of the studies

**Figure 2:**
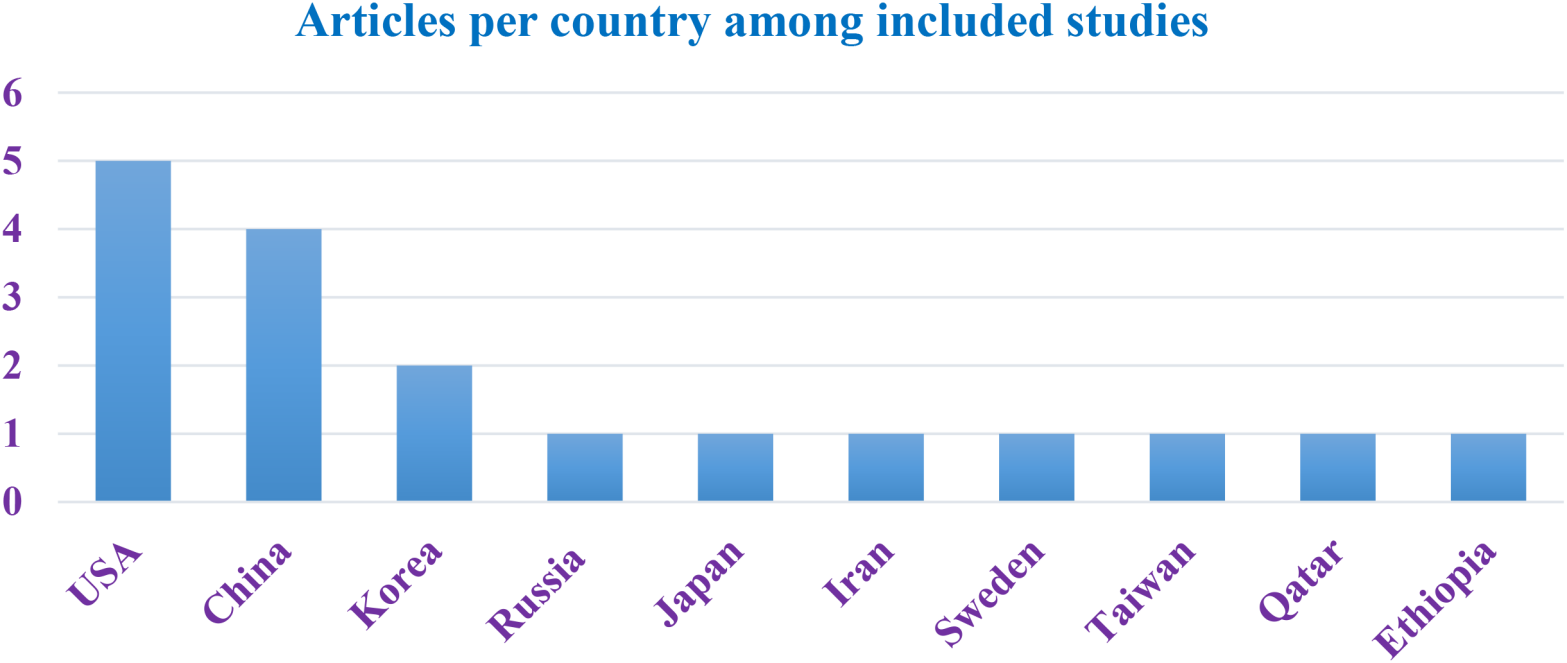
Country distribution included studies in the integrative review on sleep duration, sleep pattern, insomnia and its association with metabolic syndrome.

All the eligible studies were published between 2010 and 2024 and were conducted from ten different countries, including United States of America (n=5), (46), China (n=4) (19, (43)), Korea (n=2) (37), Russia (n=1) (53), Japan (n=1) (45), Iran (n=1) (61), Sweden (n=1) (62), Taiwan (n=1) (50), Qatar (n=1) (42), and Ethiopia (n=1) (44), respectively (**Figure 2**).

Several study designs consisted of cross-sectional (n=12), Cohort (n=5), and case-control were included.

Sample size spanned from 125 subjects to 133,608 subjects, with several studies focused on both men and women. Concerning the age distribution, the study participants varied substantially across different studies. The age distribution included general adults (44), college students (43), middle aged (30–55 years) (37), older adults including postmenopausal women and retired workers (48, 52). Some studies emphasized specific working groups such as health care providers and shift workers (40, 61).

**Figure 3:**
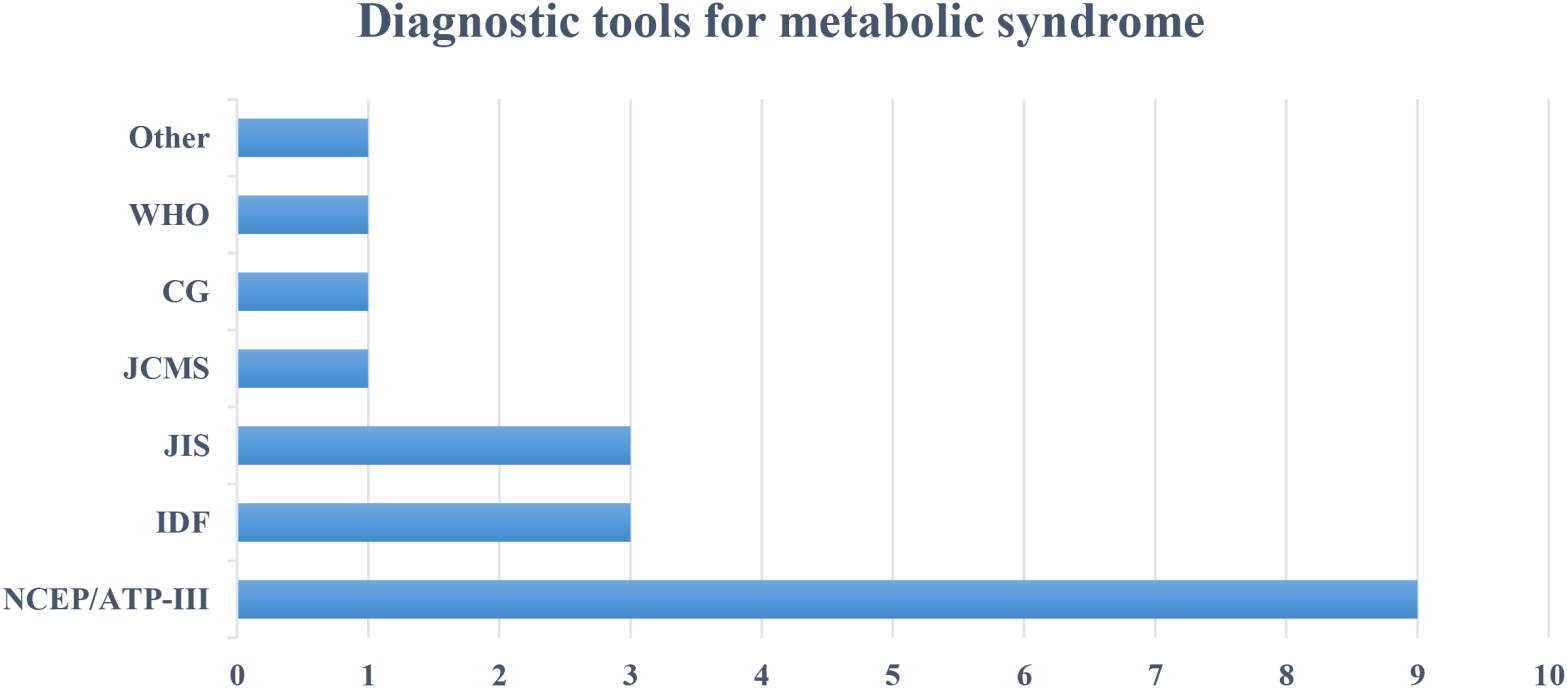
Comparison of diagnostic criteria used in the included studies in the integrative review on sleep duration, sleep pattern, insomnia and its association with metabolic syndrome. (NCEP/ATP-III, National Cholesterol Education Program/Adult Treatment Plan; IDF, International Diabetic Federation; JIS, Joint interim summit; JCMS, Japanese Criteria for Metabolic Syndrome; CG, Chinese Guideline).

MetS was diagnosed by using IDF (n=2) (51, 52, 63), NCEP/ATP-III (n=9) (18, 37, 40, 46, 49, 53, 61, 62), WHO (n=1) (42), Joint Interim Statement 2009 (JIS) (n=3) (44, 48, 50), Japanese Criteria for Metabolic syndrome (JCMS) (n=1) (45), Chinese guideline (CG) (n=1) (19), and other unspecified definition criteria (n=1) (43, 64) (**Figure 3**).

The majority of studies assess sleep duration and sleep pattern through self-reported questionnaires, while a few number of studies employed a validated Pittsburgh Sleep Quality Index (PSQI) (37, 43).

Nearly half of the eligible studies reported insomnia with a various Insomnia measurement tools, including Women Health Initiative Insomnia Rating Scale (WHIIRS) (46), Insomnia Severity Index (ISI) questionnaires (43, 61, 62), Athens Insomnia Scale (AIS) (51), Insomnia Symptom Questionnaire (ISQ) (53) and America Psychiatric Association (APA) (50).

Social Jetlag was also reported by one study and was defined as the absolute difference between mid-point sleep duration in weekdays and weekends. It was categorized as normal social jetlag (less than 1 hour), moderate social jetlag (1 hour to less than 2 hour) and sever social jetlag (2 hours and more) (40).

### 3.3 Association between sleep duration and metabolic syndrome

The majority of studies reported that short sleep duration consistently was associated with MetS (18, 37, 41, 42, 44, 50, 61). People who had low sleep duration had an increased risk of developing metabolic risk. In Some studies, short sleepers have a 2 to 5 times increased odds of developing MetS compared to normal sleep duration (42, 44). Another study conducted on premenopausal and postmenopausal women found that short sleep duration was highly prevalent in both groups with a high proportion of MetS observed in premenopausal women (48).

A few studies identified a U-shaped association between sleep duration and MetS (19, 49). It indicates that both short sleep duration and long sleep duration were linked with the increased odds of MetS. Hence the lowest risk of MetS was observed in people sleeping on average 7 hours/night (49).

Long sleep duration greater than or equal to 8 hours on average was founded to have a mixed association with MetS, indicating both increased and decreased risk for the outcome. In a few studies long sleep (greater than or equal to 8 hours on average)was associated with decreased risk of MetS (44, 50), while in others studies MetS was higher among long sleepers (18, 40, 41, 46, 52).

### 3.4 Association between insomnia and metabolic syndrome

Several studies found both specific insomnia symptoms and syndromic definition of insomnia linked with increased risks of MetS (46, 65). In the longitudinal analysis of a follow-up study change from insomnia to restful sleep overtime was associated with increased risk of MetS (46). Another study also reported high prevalence of MetS among health care workers experienced insomnia compared to those without insomnia symptoms (61). Similarly, both premenopausal and post-menopausal women with insomnia symptom were significantly associated with MetS, and the outcome was stronger among premenopausal women compared to post-menopausal women (48). Additionally, individuals with insomnia had a two-fold increased risk of MetS compared to those without insomnia (62).

Finally, insomnia was not independently associated with MetS across all gender and age groups, since the association between insomnia and MetS was statistically significant in the male group and the middle-aged group but not in the female group, the young adult group or the older group (51).

### 3.5 The association between specific sleep characteristics (patterns) with metabolic syndrome

In different studies long sleep latency, early wake-up, longer duration daytime napping (> 90 minutes), irregular sleep and social jetlag were found to have increased risk for MetS. Among individuals without MetS at baseline, long sleep latency > 30 minutes and early wakeup time in the morning showed a significant increase in MetS risk overtime (37). In another study population, female participants who napped longer than 90 minutes during the day had high prevalence of MetS relative to those who didn’t nap (52).

**Figure 4:**
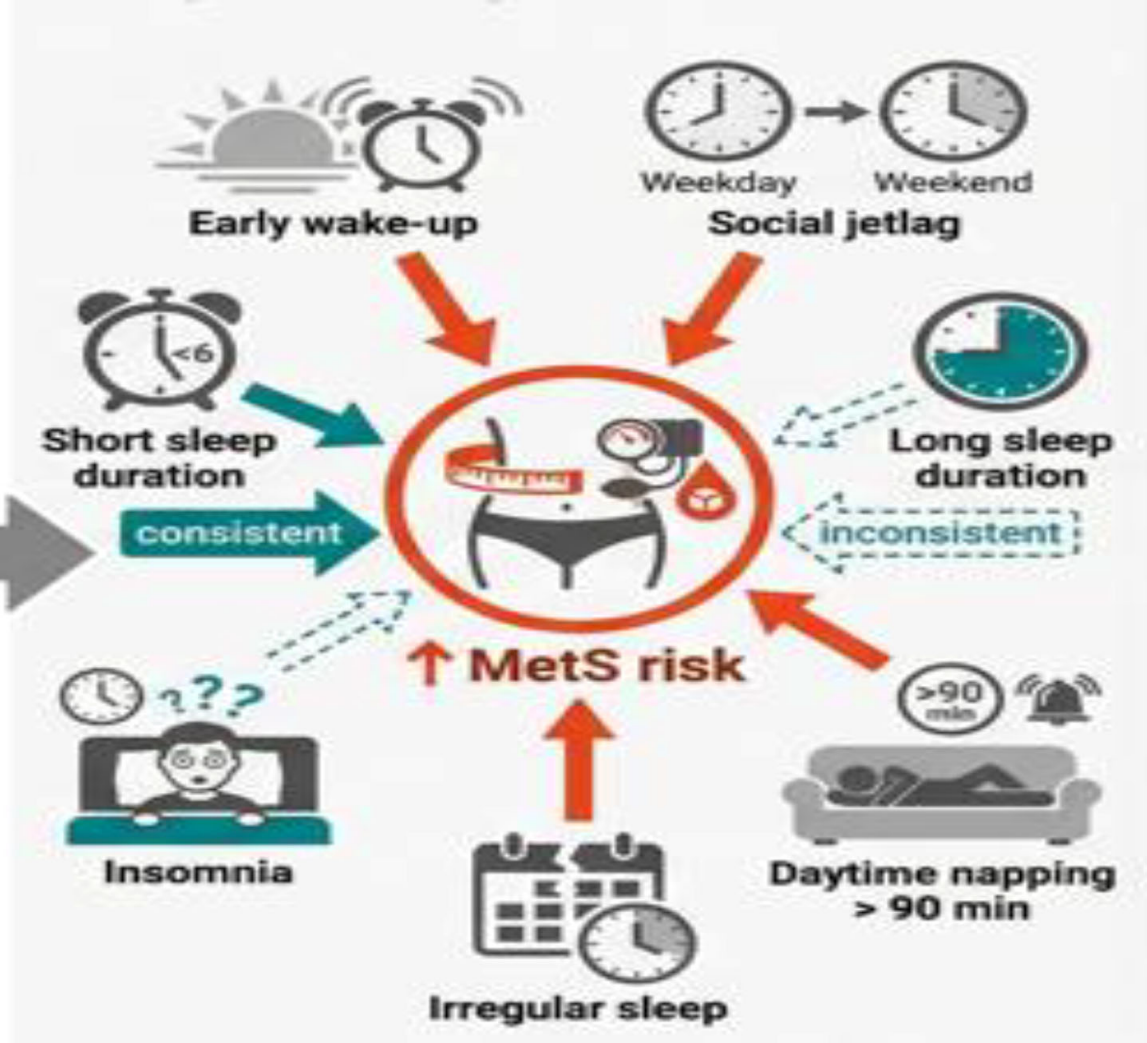
Sleep characteristics and risk of metabolic syndrome.

A behavioral cohort study involving college students showed that poor sleep pattern was significantly associated with higher metabolic scores at two-year follow-up period (43). A study on the subjective sleep irregularity showed that irregular sleep was strongly associated with MetS compared to sleep duration (45). Moderate social (1 hour-< 2 hours) showed protective effect, although its interaction with short sleep duration was associated with an increasing metabolic risk (40) (**Figure 4**).

## 4 Discussion

In this integrative review we synthesized quantitative evidences from diverse methodologies including; cross-sectional, case-control, and cohort studies. The review examined the association between sleep duration, insomnia and sleep pattern with MetS.

This review revealed that short sleep duration was consistently associated with increased odds of MetS. Several studies reported sleeping fewer than 6 hours/day significantly associated with increased odds of MetS (18, 37, 40–42, 44, 50, 63). The reasons for short sleep duration include long working hours, house and childcare responsibilities in working parents and early morning awakening, increased use of Technology in evening with sleep delayed (66). The findings supported by scientific evidences suggesting that insufficient sleep may cause reciprocal changes in circulating levels of leptin and ghrelin (67) which would increase appetite, caloric intake, reduce energy expenditure facilitating an increase in waist circumference as well as overall obesity development (38).

Short sleep duration has been linked to a number of intermediary pathophysiological processes, including alterations in the autonomic nervous system (ANS) with global sympathetic hyperactivity (23, 68, 69), inflammatory responses (70, 71), endothelial dysfunction (72), and atherosclerosis (73). These pathways are all significant risk factors for the emergence of metabolic diseases (74, 75).

In addition, few studies have shown a U-shaped association between sleep duration and MetS, where both short and long sleep duration were correlated with the outcome. While there is a definite pathophysiologic connection between short sleep duration and MetS, comorbidity and residual confounding factors may account for the correlation between lengthy sleep duration and MetS. Unrecognized factors such as sleep fragmentation, exhaustion and depression could lead to both MetS and an increased desire for sleep (76). This link may be explained by the fact that long sleepers have less time during the day to engage in physical activity (77, 78). This indicates that a moderate or physiologically appropriate sleep duration is important for metabolic health. When sleep duration is higher or lower than this range, the risk of MetS increases.

Several studies showed that insomnia and specific symptoms of insomnia were significantly associated with increased risk of MetS (46, 62). Insomnia is a subjective experience of difficulty initiating sleep, difficulty maintaining sleep, waking up too early, non-restorative sleep or poor quality of sleep (79). Many people increasingly affected by insomnia due to increased pressure from work and life with economic and society development (80, 81). In this review, although the longitudinal study employing GEE (generalized estimated equation) showed the change from restful sleep to insomnia was significantly associated with the increased odds of MetS (46), the lack of further mediation analysis prevented the establishment of the causal pathways, therefore it should be interpreted as average effect rather than causal estimate (82).

A number of studies linked insomnia and metabolic syndrome through a complex mechanism, including immune, neuroendocrine and metabolic pathways. A gut-brain axis, the communication network, where the gut microbial, gastrointestinal tract and brain regulate the sleep wake cycle. Hence, alteration in gut microbiota composition, affect both metabolic process and sleep behavior (83). Another mediating factor between insomnia and metabolic syndrome is obstructive sleep apnea (OSA). Obstructive sleep apnea often comorbid with insomnia and facilitate the risks of metabolic syndrome through intermittent hypoxia, which activates sympathetic nervous system, pancreatic B-cell dysfunction, adipose tissue inflammation and insulin resistance (84). Additionally, circadian misalignment following bad sleep habits promote the development of metabolic syndrome through disruption of hormones that regulate appetite (85).

Nevertheless, there are studies that showed only specific symptoms, but not insomnia were associated with Mets (48, 53). Therefore, although an association has been observed, whether insomnia is associated with MetS or not, is yet not conclusive.

Additionally, individual sleep characteristics (patterns) including, long sleep latency (37), early wake time (37), long duration daytime napping (52) and irregular sleep (45) were significantly associated with MetS. Longer daytime napping disrupt circadian rhythm, potentially causing metabolic and endocrine abnormalities (86). The underline biological pattern is unclear, but several possible explanations have been proposed. Longer napping may increase sympathetic activity lead to activation of the renin angiotensin system, disrupt glucose regulation, elevate cortisol levels and increased fat accumulation (87). Irregular sleep, characterized by a high night to night variability in sleep duration and timing, could lead to mild disruption of circadian rhythm, consequently increase cardio metabolic risk (88). Prospective studies showed that difficulty of falling sleep (Long sleep latency) has been linked with increased metabolic severity scores and elevated fasting blood glucose levels, indicating delayed sleep onset contributing to the development of MetS (89). Early wake-up times together with insufficient sleep and circadian miss alignment has also been reported to be potential contributing factors for MetS (90).

Among non-shift workers, 1–2 h of social jetlag was linked to a reduced risk of MetS after additional adjustment for the interaction between shift work and social jetlag (40). Social jetlag’ refers to a misalignment of sleep timing between work and free days (91),however studies show that individuals with ≥2 hours difference in sleep timing between working days and free days had double risk of metabolic syndrome and central obesity (92). Social jetlag also affects fat metabolism by disrupting prolactin hormone secretion, which control hepatic lipid metabolism (93). Additional evidences have also witnessed that social jetlag has been associated with (late meal intake, high calories food intake) (94), microbiota composition (95) and increased body mass index (96),all of which partially mediate the development of metabolic syndrome.

### 4.1 Strength and limitation of the study

One of the great strength of this review is that it allows us to combine findings from multiple designs including (Cross-sectional, cohort and case control). In addition, it helps us review gaps in literature by observing inconsistencies. Despite this, it is important to acknowledge limitations such as; the review included only literatures published in English, where missed articles would have a significant improvement in understanding sleep and Mets. Additionally, majority of the studies utilized self-reported sleep duration and insomnia questionnaires, this might introduce measurement bias and could have impact on the over report. Furthermore, in this integrative review we have synthesized predominantly cross-sectional studies, therefore it was not feasible to determine the causal relationship between different sleep characteristics with metabolic syndrome.

## 5 Conclusion

In this integrative review we synthesized evidences on the link between sleep duration, insomnia and some specific characteristics with MetS. The review revealed that short sleep duration has a consistent association with increased risk of MetS. Long sleep duration has a link with the odds of metabolic syndrome in prevalent studies, but not in longitudinal studies. This indicated that relationship between long sleep and metabolic syndrome could be due to confounding factors. The relationship between Insomnia and metabolic syndrome has remained inconsistent and yet inconclusive. Several mechanisms linked the association between Social jetlag, early wake-up, long sleep latency and long daytime napping with increased risk of metabolic syndrome.

The findings of this integrative review has some significant clinical implications. First, health care providers should consider sleep as a modifiable risk factor for MetS and include it as the integral component during metabolic health assessment. Second, individualized care plan is essential to address individual patient needs. Further research, especially prospective follow-up study need to be considered to see the connection of individual metabolic components with various sleep aspects. 
